# Postpartum aortic dissection. A case report and review of literature

**DOI:** 10.1016/j.ijscr.2019.02.018

**Published:** 2019-03-06

**Authors:** Valeria Silvestri, Giuseppe Mazzesi, Rita Mele

**Affiliations:** a Surgical Science Department, Policlinico Umberto I, University of Rome “La Sapienza”, Rome, Italy; b Cardiac Surgery Unit, Cardiovascular, Respiratory, Nefrologic and Geriatric Department, Policlinico Umberto I, University of Rome “La Sapienza”, Rome, Italy

**Keywords:** Aortic dissection, Post-partum, Pregnancy, Case report

## Abstract

**Introduction:**

Aortic dissection is a rare cardiovascular complication in pregnancy. Most of the cases occur during the third trimester of pregnancy, whilst 33% of cases are reported during the postpartum period.

**Presentation of case:**

We report the case of a multiparous 35-year-old patient with gestational hypertension treated for a type A aortic dissection on the second postpartum day. A review of literature on non-syndromic sporadic aortic dissection during the postpartum period is presented.

**Discussion:**

Aortic complications in pregnancy have been described in genetic syndromes or congenital aortic malformations but may also be non -syndromic and occur in the absence of any other risk factor. Pregnancy carries a 25-fold increase in relative risk for dissection. A review of the 16 cases published in literature from 1995 to December 2016 of non-syndromic, sporadic aortic dissections in pregnancy showed that the event may occur more frequently in the first week post-partum, be symptomatic for thoracic pain or dyspnoea. Type A aortic dissection accounts to 75% of cases. Mortality, despite surgical treatment, has been reported in 4 cases.

**Conclusions:**

Even though rarely reported, given the increasing incidence and the high mortality of aortic dissection in pregnancy, along with the potential challenge for two lives, clinician must consider aortic dissection in post-partum while dealing with differential diagnosis in post-partum patients in the emergency setting.

## Highlights

• Cardiovascular comorbidities may complicate pregnancy.
• Pregnancy carries a 25-fold relative risk for aortic dissection.
• Dissection may occur in pregnancies without known genetic or anatomical risk factors (non syndromic sporadic aortic dissection).
• Dissection may occur in the postpartum period.
• Given the high mortality for both mother and foetus, a high clinical suspicion for aortic dissection is needed in an emergency setting in postpartum.

## 1 Introduction

Different cardiovascular morbidities may complicate pregnancy, and incidence of these events is increasing in recent years. Among these conditions, acute aortic dissection has been described in pregnancy, with a reported relative risk of 25 folds. Aortic dissection is the most common cause of maternal cardiovascular death [1] and it has been described previously in patients without risk factors [2].

We report a case of a non syndromic sporadic post partum aortic dissection in a 35 year old patient which was successfully managed and followed up at our University Hospital Cardiothoracic surgical Unit, after emergency transferal from a peripheral hospital, giving details on presentation, surgical treatment and condition at 10 years follow up. A review of literature of cases of sporadic non syndromic aortic dissection has been added, focusing on risk factors, symptoms at onset, type of lesion, details on surgical management and maternal and foetal outcome.

The work has been reported in line with the SCARE criteria [3].

## 2 Presentation of case

A 35-year-old Caucasian woman was brought to the emergency department of a peripheral hospital because of excruciating chest pain, described as a sudden, stubbing retrosternal pain irradiated to the back. Two days earlier the patient underwent caesarean delivery. Her pregnancy was complicated by moderate hypertension, but without any other comorbidity. Diagnostic algorithm for acute thoracic pain included a computed tomography, which showed a type A aortic dissection with the intimal tear in the aortic root. The dissection involved the entire aorta and both the common iliac arteries. The celiac trunk as well as the mesenteric and renal arteries originated from the true lumen of the aorta. The patient was transferred by helicopter to our department, where she arrived hemodynamically stable, eupnoic, with a blood pressure of 150/70 mmHg, a heart rate of 62 bpm and a SO2 of 97%. Exams showed mild anemia (110 g/dl), with no signs of ischemic or visceral ischemia. The patient underwent emergency surgery with a supra-coronary replacement of the ascending aorta. Median sternal access and pericardiotomy exposed an acutely dilated ascending aorta compatible with acute dissection, and an hypertrophic myocardial wall as for chronic hypertension. An intimal aortic tear was found at 2 cm from the aortic valve plane. Ascending aorta was replaced with interposition of a tubular Dacron graft (Intergard 24) and suspension of aortic commissures. Cerebral perfusion was maintained through right subclavian artery. Intermittent cold blood cardioplegia was obtained directly through coronary ostia and indirectly through coronary sinus. Warm blood reperfusion preceded declamping.

Recovery was uneventful and patient was discharged in good conditions nine days after surgery. Thoracic abdominal angio-CT scan, routinely performed for post-operatory control, showed uncomplicated ascending aortic graft, residual dissection from distal anastomosis extended to both common iliac arteries, down to right external iliac artery and left internal iliac artery. Celiac tripod, superior and inferior mesenteric and both renal arteries were perfused by true lumen.

Marfan syndrome was excluded by genetic tests.

**Fig. 1:**
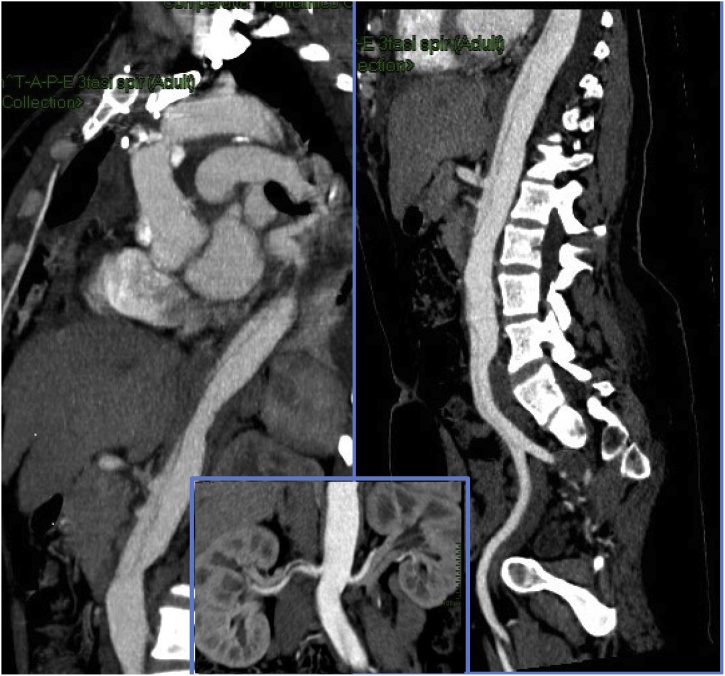
Post operatory images after ascending aortic substitution with tubular graft on the left. Residual aortic dissection through descending thoracic and abdominal aorta can be seen. Renal perfusion detail in the square below is added. Mesenteric perfusion from the true lumen can be noticed.

**Fig. 2:**
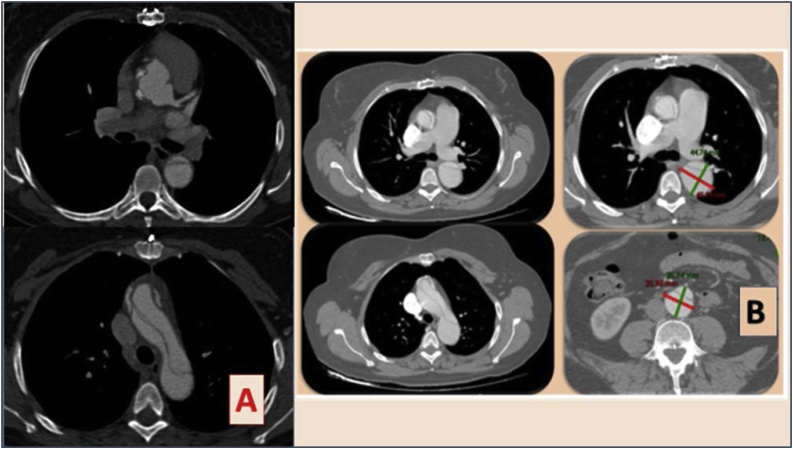
A Pre-Operatory CT scan- aortic valve replacement. B Follow up CT scan, 10 years from aortic dissection.

One and a half years later, an asymptomatic aortic valve regurgitation with an enlarged left ventricle chamber (68 mm × 50 mm; volume of 93 cm 3) was detected at echocardiography. Pre-operatory assessment was integrated with CT scan [Fig. 2]. The patient underwent replacement of aortic valve with a mechanical valve prosthesis (St Jude Regent 21) and the recovery was uneventful. CT scan performed before discharge showed aortic valve in place, aortic bulb of 42 mm, a stable aortic arch dissection extending to the descending thoracic and abdominal aorta as well as the iliac arteries, with visceral perfusion through true lumen [Fig. 1]. At 10 years, the abdominal aorta was found to be dilated (35 mm in diameter). Aortic bulb was stable in its diameters on CT scan follow up [Fig. 2] No indication for further surgical treatment has been reached up until now. Patient is under regular computed tomography follow-up. Not only she has given consent for reporting her case, but has participated with enthusiasm sharing her clinical reports, her personal memories of the event and participating to additional check ups, sure that her experience may be useful to increase awareness of a rarely reported condition that may affect women in the most delicate stage of their life.

## 3 Discussion

An increased prevalence of cardiovascular morbidities complicating pregnancy has been reported in a recent retrospective Japanese study [1]. Among these, a 25-fold increase in the risk for aortic dissection in pregnancy has been previously reported in literature [4]. Indeed, aortic dissection has been reported as the most common cardiovascular cause of maternal death [1].

A Swedish study reported on an incidence of aortic rupture during pregnancy of 14.5/1 000 000, whilst its rate in non-pregnant women was 1.24/1 000 000. Furthermore, the reported case-maternal fatality ratio for aortic rupture in pregnancy was 4.4/1 000 000 [4].

An increased incidence of aortic dissection during pregnancy has been also reported in an English administrative population study, analysing England's national hospital-level data [5].

Marfan, Ehlers-Danlos, Loeys-Dietz and Turner syndrome, or bicuspid aortic valve, are conditions associated with an increased risk for aortic dissection, but non-syndromic cases have also been described in literature. Even though in some of these cases a familiarity for dissection may be reported, sporadic cases of non syndromic aortic dissections may occur [6]. Specifically for pregnancy, aortic dissection has been described previously in patients without risk factors [2]

Hemodynamic and hormonal changes in the arteries may increase the wall tension and intimal shear forces on the aortic medial layer making it more susceptible to injury during pregnancy [7].

Furthermore, advanced maternal age secondary to a widespread of assisted reproductive technology further increase the risk for pregnancy related hypertension and, thus of aortic dissection [8].

Angiogenic factors required for foetus and placental vascular development may cause maternal aortic remodelling and previous studies have suggested their role in the physiopathology of cardiovascular complications in pregnancy, such as hypertension and eclampsia [9]. Additionally, hormonal factors are important in remodelling process. Oestrogens may promote the increase in aortic diameter by causing structural changes, such as reticulin fragmentation and elastic fibre disorganization that lead to structural weakening of the aortic media. These changes may be more pronounced in the third trimester and in the postpartum period.

Although aortic dimensions revert toward baseline over the first 6 months postpartum, an enduring increase in aortic diameter can occur in multiparas [10]. Specifically our patient was multipara, and had four pregnancies before the last, which was complicated by aortic dissection.

As for timing, while more than 50% of dissections in pregnancy occur in the third trimester, only 33% occur during the postpartum period, as in our case.

Aortic dissection in pregnancy causes significant maternal and foetal mortality, as high as 30% and 50%, respectively [10] and this is also due to difficulty in managing differential diagnosis.

There is a broad list of described initial presentation for aortic dissection in pregnancy. Data from a cohort of 25 patients described by Zhu et al have reported abrupt onset of sharp chest or back pain radiating to the neck or shoulders as a predominant symptom (80% of cases). Syncope, dyspnoea or orthopnoea, but also asymptomatic presentation have been described [11].

As recommended by the European society of cardiology guidelines for the management of cardiovascular disease during pregnancy, any pregnant woman presenting with acute chest pain must be considered for aortic dissection, alongside myocardial infarction, preeclampsia and pulmonary embolism [12].

We briefly reviewed literature in order to find other cases of aortic dissection in the postpartum period, in patients without previously known genetic risk factors (Marfan syndrome, other connective tissues disorder, Turner syndrome). We retrieved 16 cases. Mean age of patients was 31.8 ± 5.2. Aortic dissection occurred more frequently in the first week post-partum (11 cases, 66%). In the majority of patients, no risk factor was reported (10 cases, 62%). When risk factors were present, hypertension was the most frequently reported (4 cases, 25%), followed by eclampsia, pre-eclampsia, obesity and twin pregnancy. Thoracic pain was the most common symptom referred on presentation (7 cases, 43.8%), but dyspnoea, abdominal pain, cardiogenic shock, loss of consciousness, lower limb hyposthenia, pulmonary oedema or sudden death have also been described as main clinical presenting features. Interestingly in four cases [[13], [14], [15], [16]] a previous episode of thoracic or abdominal pain during the last stages of pregnancy usually preceded the postpartum event, suggesting that in some cases a strong clinical suspicion for a cardiovascular complication could permit a more accurate patient management. Specifically, in the case reported by Bjornstad, an organized clot was found on pericardial exploration, with effusion of some age, maybe occurring during pregnancy, unfortunately undiagnosed before acute onset [15]. As for treatment, the majority of cases underwent open surgery (8 cases, 50%), followed by endovascular or hybrid approach. Two cases haven’t been treated, because of refusal in one case, and sudden death in another. Maternal mortality was reported in two other cases (4 cases, 25%). In one case death was due to retrograde dissection after surgical treatment [17] while in the other, in which aortic dissection was associated with anterior myocardial infarction, maternal exitus was due to severe post operatory myocardial dysfunction [18].

**Fig. 3:**
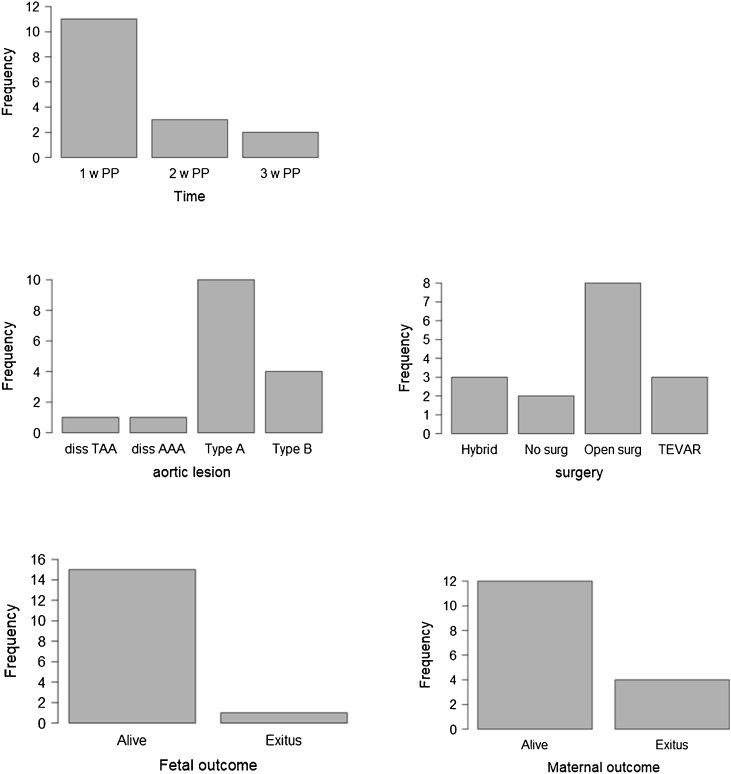
Histograms resuming data on timing of presentation, type of dissection, surgical options, maternal and foetal mortality. Frequency refers to number of cases on a total of 16.

Data from cases have been summarized in Table 1. Cases have been singularly reported in Table 2 [[13], [14], [15],[18], [19], [20], [21], [22], [23]]. Histograms have been built to resume data on timing of presentation, type of dissection, surgical options and maternal mortality [Fig. 3].

**Table 1:** Descriptive statistics of data form case reports. number of cases and corresponding % of total cases have been specified.

Descriptive statistical analysis of age, timing of event, risk factors for dissection, symptoms at onset, type of lesion, surgical management and maternal and foetal outcome (%)		
**Age**	31.8 ± 5.2	
	N cases	%
**Timing**		
I w PP	11	68.8
2 w PP	3	18.8
3 w PP	2	12.5
**Risk factors**		
No risk factors	10	62.5
Hypertension	4	25
Eclampsia/preeclampsia	2	12.5
Obesity	2	12.5
Twin pregnancy	1	6.3
**Presentation**		
Thoracic pain	7	43.8
Dyspnea	2	12.5
Abdominal pain	1	6.3
Cardiogenic shock	1	6.3
Loss of consciousness	1	6.3
Lower limb hyposthenia	1	6.3
Pulmonary edema	1	6.3
Sudden death	1	6.3
**Type of lesion**		
Type A aortic dissection	10	62.5
Type B aortic dissection	4	25.0
Dissected thoracic aortic aneurysm	1	6.3
Infrarenal dissected aneurysm	1	6.3
**Treatment**		
Open surgery	8	50.0
TEVAR	3	18.8
Hybrid	3	18.8
No surgery	2	12.5
**Maternal outcome**		
Alive	12	75
Exitus	4	25
**Fetal outcome**		
Alive	15	93.8
Exitus	1	6.3

**Table 2:** Previously published cases of non syndromic aortic dissection in pregnancy. Details on risk factors, dissection type, symptoms at onset, surgical management and maternal and foetal outcome are given.

Author Year	Age	Risk factors	Time	Presentation	Aortic lesion	Surgery	Hystology	Maternal Outcome	Fetal outcome
Kahil 1995 [12]	26	Hypertension in second trimester; twin pregnancy	2 w PP	Abdominal pain	4 cm dissected infrarenal aortic aneurysm	Aorto-iliac bypass graft	Perivascular lymphocytic vasa vasorum infiltration; elastic fibers loss	Alive	Alive
Omar 2007 [17]	30	None	1 w PP	Acute severe retrosternal chest pain diaphoresis	Type A aortic dissection + left coronary artery + NSTEMI	Bental procedure	Cystic medial necrosis	Exitus	Alive
Savi 1 2007 [13]	38	Obesity	1 w PP	Thoracic pain pulmonary edema	Type A aortic dissection left coronary and descending thoracic aorta	Ascending aortic substitution; right coronary sinus repair	Cystic Medio necrosis, elastic fiber shearing	Alive	Alive
Savi 2 2007 [13]	33	None	1 w PP	Sudden left leg hyposthenia dyspnea	Acute type A aortic dissection on 55 mm ascending aorta- left atria to celiac trunk	Ascending aortic substitution and subcomissural valvuloplasty	Mucoid imbibition – Elastic fiber shear	Alive	Alive
Bjornstad 2008 [14]	22	None	1 w PP	Cardiogenic shock	Type A + right coronary; cardiac tamponade	Ascending aorta bypass; aortic valve and coronary reimplantation	None	Alive	Alive
Monteiro 2011 [18]	26	None	1 w PP	Sudden death	Dissected saccular aneurysm descending thoracic aorta (below istmus)	–	Myxoid degeneration, elastic fibers distruction,intimal+ adventitial chronic aortitis	Exitus	Alive
Rosenberger 2012 [19]	30	Eclampsia with fetal demise 4 days before, obesity, hypertension	1 w PP	Severe chest + abdominal pain	Type b aortic dissection, descendant thoracic rupture, mesenteric ischemia	TEVAR + distal extension above celiac trunk 2 days later for increased filling of false lumen	None	Alive	Exitus-vaginal demise y 1 w before event
Jalalian 2013 [15]	34	None	3 w PP	Dyspnea respiratory distress for 2 days	Cardio-myopathy + type A aortic dissection	Hybrid thoracic abdominal reconstruction	None	Alive	Alive
Yang 2014 pt 3 [20]	35	None	2 w PP	Thoracic pain	Type A aortic dissection	Refused surgery	None	Exitus	Alive
Yang 2014 pt 5 [20]	39	None	3 w PP	Chest pain	Type A aortic dissection	Aortic graft + TEVAR + CACG	None	Alive	Alive
Yang 2014 pt 7	26	None	1 w PP	Chest and back pain	Type A aortic dissection	Bentall	None	Alive	Alive
Yang 2014 pt 8 [20]	29	Hypertension	1 w PP	Chest and back pain	Type B aortic dissection	TEVAR	None	Alive	Alive
Yang 2014 pt 11 [20]	36	Hypertension	2 w PP	Chest pain and dyspnea	Type B aortic dissection	Aortic graft + TEVAR	None	Alive	Alive
Shu 2014 [21]	31	None	Min after labor	Sudden chest pain and loss of consciousness	Type B aortic dissection	TEVAR	None	Alive	Alive
Esteves 2016 [22]	35	Pre eclampsia 34 w pre-term delivery	Min after labor	Severe acute thoracic pain, nausea, sweating	Type A aortic dissection / aortic root-celiac trunk	Aortic root replacement with tubular graft	None	Alive	Alive
Yalcin 2016 [16]	40	None	1 w PP	Severe chest and back pain	43 mm ascending aorta dilatation-type A aortic dissection	Ascending aorta replacement complicated by retrograde dissection	None	Exitus	Alive

In one case foetal death was reported, one week previously to aortic dissection, in a patient suffering from eclampsia. Foetal death was reported in no other case.

## 4 Conclusions

Non-syndromic aortic dissection may occur during pregnancy or in the postpartum period with an incidence of 14/1,000,000, which has been reported to be increasing recently by several population based studies [1,4,5]. Even though rarely reported, aortic dissection may be sporadic, and may occur in patients with no reported familiarity or anamnestic risk factors for aortic dissection. Differential diagnoses in these cases may be challenging, leading to treatment delay. Acute aortic dissection is usually linked to a high mortality, with a fatality ratio of 4.4 / 1,000,000 for aortic rupture reported specifically in pregnancy contest [4]. Even though a reduction in surgical mortality rate for dissection has been reported in recent studies, it is still high, reaching a mortality of 18% according to International Registry for Acute Aortic Dissection (IRADD) data [24]. Non syndromic sporadic aortic dissection in pregnancy has been reported rarely in literature and, apart from our case, 16 reports have been retrieved in literature. Still, given the high mortality of this condition and the potential challenge for two lives, clinician must consider aortic dissection in post-partum patients while conducting differential diagnosis in emergency setting, thus preventing delay in treatment, which is linked to a poor outcome.

## Conflicts of interest

No conflict of interest or financial disclosure must be added

## Sources of funding

No sources of funding have been used for the submitted work

## Ethical approval

No ethical approval needed Ethical approval has been exempted by your institution.

## Consent

Written informed consent was obtained from the patient for publication of this case report and accompanying images. A copy of the written consent is available for review by the Editor-in-Chief of this journal on request

## Author contribution

The authors have contributed equally to the submission

Dr Valeria Silvestri has carried out literature revision as a first reviewer; Professor Mele was responsable of the patient follow up planning and of acting as second reviewer of literature; Prof Mazzesi was responsible of the surgical case and has provided clinical and surgical details for the case. All authors have contributed to draft writing and revision.

## Registration of research studies

Not needed

## Guarantor

Valeria Silvestri.

## Provenance and peer review

Not commissioned, externally peer-reviewed.
